# Fully Human Antagonistic Antibodies against CCR4 Potently Inhibit Cell Signaling and Chemotaxis

**DOI:** 10.1371/journal.pone.0103776

**Published:** 2014-07-31

**Authors:** Urs B. Hagemann, Lavinia Gunnarsson, Solène Géraudie, Ulrike Scheffler, Remko A. Griep, Herald Reiersen, Alexander R. Duncan, Sergej M. Kiprijanov

**Affiliations:** Affitech Research AS, Oslo, Norway; National Cancer Institute, NIH, United States of America

## Abstract

**Background:**

CC chemokine receptor 4 (CCR4) represents a potentially important target for cancer immunotherapy due to its expression on tumor infiltrating immune cells including regulatory T cells (T_reg_s) and on tumor cells in several cancer types and its role in metastasis.

**Methodology:**

Using phage display, human antibody library, affinity maturation and a cell-based antibody selection strategy, the antibody variants against human CCR4 were generated. These antibodies effectively competed with ligand binding, were able to block ligand-induced signaling and cell migration, and demonstrated efficient killing of CCR4-positive tumor cells via ADCC and phagocytosis. In a mouse model of human T-cell lymphoma, significant survival benefit was demonstrated for animals treated with the newly selected anti-CCR4 antibodies.

**Significance:**

For the first time, successful generation of anti- G-protein coupled chemokine receptor (GPCR) antibodies using human non-immune library and phage display on GPCR-expressing cells was demonstrated. The generated anti-CCR4 antibodies possess a dual mode of action (inhibition of ligand-induced signaling and antibody-directed tumor cell killing). The data demonstrate that the anti-tumor activity *in vivo* is mediated, at least in part, through Fc-receptor dependent effector mechanisms, such as ADCC and phagocytosis. Anti-CC chemokine receptor 4 antibodies inhibiting receptor signaling have potential as immunomodulatory antibodies for cancer.

## Introduction

The G-protein coupled chemokine receptors and their ligands, the chemo-attractant cytokines or chemokines, play crucial roles in both homeostasis and disease [1]. The chemokine receptors are also involved in a wide variety of pathological inflammatory and immune responses through chemo-attraction of innate and adaptive immune cells. Their homeostatic roles include the leukocyte maturation and trafficking, organogenesis, angiogenesis, and tissue repair [2]. In cancer, the chemokines and their receptors are responsible for trafficking of immune and tumor cells into and out of the tumor microenvironment [3]. For example, the aberrant expression of the chemokine receptors on tumor cells can promote tumor metastasis in the secondary organs that release the corresponding chemokine ligands [4].

CCR4 and its ligands, the thymus and activation regulated chemokine (TARC/CCL17) and the macrophage-derived chemokine (MDC/CCL22), play a key role in development and progression of solid tumors through orchestrating the recruitment and trafficking of immune cells, including the immunosuppressive FoxP3^+^ CD25^+^ CD4^+^ regulatory T cells (T_reg_) into the lymphoid infiltrates surrounding the tumor [5]–[7]. As a mechanism of T_reg_ recruitment to tumors, it has been proposed that the tumor cells and tumor infiltrating macrophages produce the chemokine CCL22, which attracts and recruits CD25^+^ CD4^+^ T_reg_s expressing CCR4 [8], [9]. The T_reg_ cells can inhibit tumor-specific immunity through a variety of contact-dependent and contact-independent mechanisms and their increased numbers in tumors and draining lymph nodes correlate with poor prognosis in several types of cancer, including cancers in head and neck, lung, liver, gastrointestinal tracts, pancreas, breast or ovary [10], [11]. Studies in mouse disease models and clinical trials demonstrate that reducing T_reg_ activity boosts endogenous anti-tumor immunity and increases the efficacy of active immune interventions [12].

The CC-chemokine receptor 4 (CCR4) is also highly expressed on tumor cells of T-cell derived variants of non-Hodgkin’s lymphoma (NHL), such as adult T-cell leukemia/lymphoma (ATLL) [13], [14], cutaneous T-cell lymphoma (CTCL) [15], [16], and other kinds of malignancies belonging to the heterogeneous group of peripheral T-cell lymphoma (PTCL) [17]. In Western countries, PTCL accounts for 15–20% of aggressive lymphomas and 5–10% of all NHL [18]. PTCL remains extremely difficult to treat; most PTCL subtypes become refractory to chemotherapy regimens and relapse [19]. Among the various entities of PTCL, ATLL harbors the worst prognosis, with a 5-year overall survival (OS) and failure-free survival (FFS) of 14% and 12%, respectively [18].

During the last fifteen years, monoclonal antibodies (MAbs) have become a major immunotherapeutic modality for treatment of hematological malignancies and solid tumors [20]–[22]. The vast majority of these approved anti-cancer MAbs target surface antigens expressed on tumor cells. A number of modes of action have been described. The antibodies can induce tumor cell death by blocking the ligand-receptor interactions critical for tumor growth and survival. In addition, MAbs mediate immune effector mechanisms via their Fc portion upon binding to Fc receptors (FcR) on effector immune cells. These effector mechanisms include antibody-dependent cellular cytotoxicity (ADCC), complement-dependent cytotoxicity (CDC) and the antibody-dependent cellular phagocytosis (ADCP). An alternative (or complementary) immunotherapeutic strategy consists in modulation of the anti-tumor immune responses by targeting immune cells, irrespective of tumor antigens [23]. In particular, modulation of immunosuppressive T_reg_ cells with antibodies can enhance the efficacy of cancer immunotherapy [12], [24]. The potential approaches may include T_reg_ depletion, attenuation of T_reg_ immunosuppressive functions, prevention of T_reg_ homing at the tumor sites, and exploitation of T-cell plasticity (e.g., blocking conversion of conventional CD4^+^ T cells into induced T_reg_s or reprogramming “terminally differentiated” T_reg_s toward effector T cell subsets, such as T_H_17) [25]. Therefore, the antibodies targeting the chemokine receptor CCR4 may possess dual or multiple mode of action in some cancer indications, such as targeting the CCR4^+^ tumor cells and modulation of immunosuppressive tumor microenvironment including infiltrating T_reg_ cells.

Previous approaches to generate therapeutic antibodies against CCR4 were based on humanization of the MAbs generated by immunization of mice [26], [27]. For example, a therapeutic antibody mogamulizumab (KW-0761) recently approved in Japan for treatment of ATLL [28], [29] is a humanized version of a murine MAb KM2160 which was established by immunizing mice with a peptide corresponding to N-terminal amino acid residues 2–29 of human CCR4 [30]. The humanized antibody KW-0761 is produced as a defucosylated human IgG1 and its apparent mode of action is potent ADCC activity against CCR4^+^ cells; however, no CDC activity or effects on CCR4-mediated signaling or migration has been documented [27], [31], though induction of phagocytosis has been described [32].

In the present report, we describe the generation of fully human antagonistic antibodies against CCR4 using human non-immune antibody library, *in vitro* display technology and screening on intact CCR4^+^ cells. The antibodies possess dual mode of action: potent ADCC activity against CCR4^+^ cells of different origin as well as inhibition of ligand-induced CCR4-mediated cell signaling and cell migration. In addition, cross-reactivity with the receptors from both non-human primates and mouse is demonstrated.

## Materials and Methods

### Ethical Statements

#### Human Samples

This work took advantage of screening an existing human antibody library. The generation of this library is described in the work of Løset *et al.* [33]. Written consent was obtained from all donors and an example of such consent has been amended during the submission process. In experiments where human peripheral blood mononuclear cells (PBMCs) have been isolated, the supply of buffy coats was from the blood bank of the Oslo University Hospital and hence under license and in accordance with the Norwegian law. Further, in agreement with the Norwegian law, all work performed by Affitech involving human samples was approved by the Norwegian ethical committee, so called Regional Committees for Medical and Health Research Ethics (REC; Oslo, Norway). The respective documents have been attached during the submission process.

#### Animal studies

Animal studies described in the manuscript comprised a xenograft model using athymic nude mice. These studies were conducted at the research facilities of Experimental Pharmacology and Oncology GmbH (EPO) in Berlin-Buch, Germany. EPO has approved animal facilities for the maintenance of rodents under barrier and pathogen-free conditions. Ethical approval has been granted by the LAGeSO (State Office of Health and Social Affairs, Berlin, Germany). The respective approval documents have been attached during the submission process. Upon reaching the humane endpoint with a tumor volume of 1,500 mm^3^, the animals were sacrificed by cervical dislocation.

### CCR4-Expressing Cell Lines

The cell lines 786-O (human renal cell cancer), CCRF-CEM (human acute T-lymphoblastic leukemia), DT-40 (chicken lymphoma), HEK-293T/17 (human embryonic kidney), HUT78 (human cutaneous T-cell lymphoma) and Renca (murine renal cell cancer) were purchased from the American Type Culture Collection (ATCC, Rockville, MD). The human Hodgkin’s lymphoma cell line L428 was obtained from the German Collection of Microorganisms and Cell Cultures (DSMZ, Braunschweig, Germany). The CCRF-CEM and DT-40 cells were maintained in RPMI-1640 culture medium and the HEK-293T cells were maintained in Dulbecco’s Modified Eagle Medium (DMEM). All cells were maintained in media supplemented with Penicillin and Streptomycin and with fetal bovine serum (FBS; PAA Laboratories, Pasching, Austria); the concentration of FBS was 10% for DT-40 and HEK-293T cells and 20% for CCRF-CEM cells. The cell media and supplements were purchased from PAA Laboratories except the chicken serum that was obtained from Sigma-Aldrich (Oslo, Norway). DT-40 and HEK-293T cells were transfected with pcDNA3.1 plasmid (Life Technologies, Carlsbad, CA) comprising synthetic human CCR4 gene using the standard procedures. The CCR4 expression levels were determined using QIFIKIT from DAKO in FACSCanto II flow cytometer (BD Biosciences, San Jose, CA).

### Human Antibody Library Screening and Selection of CCR4-Specific Binders

A phage-displayed human naïve antibody library, for which written consent from donors as well as ethical approval was received from the REC (Regional Committees for Medical and Health Research Ethics; Oslo, Norway), with diversity of 6.4×10^9^ individual clones [33] was selected and screened for CCR4^+^ cell binding. In brief, the library was first depleted by pre-incubation of 10^12^ phage particles (determined as colony forming units, cfu) with 5×10^6^ non-transfected HEK-293T cells for 1 hr at room temperature (RT). The unbound phages were retrieved by centrifugation and used for incubation with 4–5×10^6^ CCR4-transfected cells for 1 hr at RT to select CCR4 binders. The cells were gently washed several times in phosphate buffered saline (PBS; Life Technologies) supplemented with 0.1% BSA followed by centrifugation. The cell-bound phages were either competitively eluted in presence of CCR4-specific chemokines, CCL17 and CCL22 (Peprotech Nordic, Stockholm, Sweden) or eluted by incubation with trypsin (Sigma-Aldrich) or with 76 mM citric acid, pH 2.5. The eluted phages were rescued for the subsequent rounds of panning by infection of exponentially growing culture of *Escherichia coli* XL-1 Blue (Stratagene, La Jolla, CA). After three panning cycles, the phagemid vector DNA was isolated from the pooled bacterial clones and the single-chain Fv (scFv) genes were excised using *Nco*I/*Not*I restriction endonuclease digestion and re-cloned into a pHOG21 bacterial expression vector [34] for production of soluble scFv fragments comprising C-terminal cMyc and hexa-histidine (His_6_) tags. The ligated DNA was used for transformation of *E. coli* TG1 bacteria (Stratagene) and single colonies were picked into 384-well X7007 plates (Molecular Devices, Sunnyvale, CA) using a QPix robot (Molecular Devices). The recombinant antibody fragments were expressed in 384-well plates by induction with 1 mM IPTG followed by overnight incubation at 30°C. The scFv-containing bacterial supernatants were retrieved by centrifugation and transferred into new 384-well plates using TecanEvo Freedom liquid handling station (Tecan, Männedorf, Switzerland). The supernatants were mixed with a mouse anti-cMyc antibody 9E10 (Santa Cruz Biotechnology, Santa Cruz, CA), Alexa Fluor 647-labeled goat anti-mouse antibody (Life Technologies) and the cells of interest. Screening of scFv clones for binding to CCR4^+^ cells and lack of binding to CCR4^−^ cells was carried out using Fluorescent Microvolumetric Assay Technology (FMAT) on an 8200 Cellular Detection System (Applied Biosystems, Foster City, CA). Affinity maturation of the selected scFv candidates was performed using previously described chain shuffling and site-directed mutagenesis approaches [35].

### Expression and Purification of anti-CCR4 Antibodies

For initial characterization, the scFv fragments were expressed in bacteria and isolated from soluble periplasmic extracts by immobilized metal-ion affinity chromatography (IMAC) on HiTrap Ni-Sepharose columns (GE Healthcare, Uppsala, Sweden) essentially as previously described [36]. Homogeneous monomeric scFv preparations for further characterization were obtained by size-exclusion chromatography of IMAC-derived material on HiLoad 26/60 Superdex 75 column (GE Healthcare) equilibrated in PBS, pH 7.4. For generation of full-length antibody molecules, the scFv-derived V_H_ and V_L_ genes were re-cloned into the mammalian expression vectors pLNO series [37] comprising cassettes for expression of human IgG1 or murine IgG2a heavy and light chains. The derived plasmids were used for transfection of HEK-293T cells with FuGENE transfection reagent (Promega, Madison, WI). The transiently transfected cells were propagated in Nunc EasyFill Cell Factories (Thermo Fischer Scientific) and the antibodies were purified to homogeneity from the cell culture supernatants by affinity chromatography on MAbSelect protein A column (GE Healthcare) followed by gel filtration on HiLoad 16/60 Superdex 200 column (GE Healthcare) equilibrated in PBS, pH 7.4. The purified antibodies consisted mainly of monomeric fractions with presence of the high-molecular weight forms (dimers and aggregates) not exceeding 5%. For production of the ADCC-enhanced (defucosylated) antibody variants, a selective inhibitor of class I α-mannosidases, *kifunensine* (Sigma-Aldrich), was added to the culture medium at concentration 100 ng/mL, as previously described [38], [39].

### Anti-CCR4 Control Antibodies

The commercial anti-human CCR4 MAb 1G1 and anti-mouse CCR4 MAb 2G12 were purchased from BD Biosciences and BioLegend (San Diego, CA), respectively. The sequences of the variable domains of the chimeric KM3060 and humanized KW-0761 antibodies recognizing the amino acid residues 2–29 of the human CCR4 [13], [30] were retrieved from the patent literature available in open access. The corresponding genes were synthesized (Life Technologies) and cloned into the human IgG1 expression vectors pLNO [37]. The chimeric (mouse variable domains/human constant domains) and humanized antibodies were produced in transiently transfected HEK-293T cells and purified using protein A chromatography followed by size-exclusion fractionation on a Superdex 200 column, as described above. The antibody variant KW-0761 was produced as a fucose-devoid IgG1 molecule by the mammalian cells incubated in the presence of kifunensine, as described above. The in-house made CCR4-specific chimeric and humanized IgG1 variants were named KM3060var and KW-0761var (variant), respectively. As an isotype control, a previously described human antibody against a hapten 6-monoacetylmorphine (6-MAM) [40] was used. In preliminary experiments, this antibody showed neither cross-reactivity with the cells used in the present study, as demonstrated by flow cytometry, nor binding to human, mouse and monkey tissues, as demonstrated by immunohistochemistry (IHC).

### Cell-binding Experiments

The cell lines were cultured under the conditions recommended by the supplier. For flow cytometry, a total of 10^5^ cells were incubated with 100 µL PBS (Life Technologies) supplemented with 0.2% BSA and 0.09% sodium azide (Roth, Karlsruhe, Germany) (referred to as FACS buffer) and containing diluted recombinant antibodies for 60 min on ice at 4°C. After washing with FACS buffer, the cells were stained with 1 µg/mL of R-Phycoerythrin (RPE)-conjugated goat anti-human IgG (AbD Serotec, Düsseldorf, Germany) for 30 min at 4°C. The stained cells were washed and resuspended in 200 µL of FACS buffer containing 2 µg/mL propidium iodide (Sigma-Aldrich) to exclude dead cells. The fluorescence of stained cells was measured using either an EasyCyte flow cytometer (Guava Technologies, Hayward, CA) or a FACSCanto II flow cytometer (BD Biosciences). Median fluorescence intensity values were plotted against the antibody concentration and the experimental data were analyzed using a ‘one site – total binding’ equation of the software program PRISM version 5.04 (GraphPad Software, San Diego, CA).

Competitive inhibition experiments were performed either on DT40/CCR4 or CCRF-CEM cells using biotinylated IgGs at concentration 0.35–0.70 nM or biotinylated CCL17 (3.12×10^5 ^nM), which were competed for cell binding with increasing concentrations of unlabeled IgG or ligands. The cell-bound biotinylated antibodies or ligands were detected using PE-conjugated streptavidin (BioLegend). For CCL22, the ligand displacement assay was performed using in-house made Alexa-647-labeled CCL22-SNAP fusion protein at concentration 50 nM. To determine *IC*_50_ values (antibody concentrations leading to 50% inhibition of labeled protein binding), the experimental data were fitted by non-linear regression curve fit using a model ‘log [inhibitor] vs. response’ of software PRISM (GraphPad).

### Analysis of Species Cross-reactivity

HEK-293T cells, cultivated under regular conditions, were transiently transfected using FuGENE (Promega) either with pcDNA3.1 plasmid comprising synthetic gene of human CCR4 (Swiss-Prot #P51679) or with plasmids of pLNO series [37] encoding CCR4 receptor from mouse (Swiss-Prot #P51680) or from rhesus monkey *Macaca mulatta* (GenBank #AFH30272). The cells were grown for 48 hrs, washed twice with PBS and detached from the culture flasks with Accutase (PAA laboratories). The transfected cells were washed with PBS and re-suspended in FACS buffer at density of 10^6^ cells/mL. The cells from 100 µL aliquots were collected by centrifugation, re-suspended in 40 µL of FACS buffer, mixed with 10 µL antibody dilutions and incubated at 4°C for 45 min. The samples were then washed twice with PBS followed by centrifugation and re-suspension in 100 µL FACS buffer. The cell pellets were finally re-suspended in 50 µL FACS buffer comprising 3 µg/mL PE-conjugated anti-human IgG antibodies and incubated at 4°C for 45 min. The samples were washed twice as described above, re-suspended in 250 µL FACS buffer and transferred into a U-shaped 96-well plate (Corning, Schiphol-Rijk, Netherlands) for analysis on FACSCanto II flow cytometer (BD Biosciences). For binding to murine splenocytes, the splenocytes were isolated from fresh mouse spleens by filtering through a 70 µm cell strainer. The cells were washed in RPMI-1640 medium, supplemented with 10% FBS (PAA Laboratories), and collected by centrifugation at 300 *g* for 10 min at 4°C. They were then resuspended in 10 mL of red cell lysis buffer and collected by centrifugation at 300 *g* for 10 min at 4°C. The splenocytes were finally resuspended in 25 mL of RPMI-1640/10% FBS and were further activated via incubation with mouse T-activator CD3/CD28 Dynabeads (Life Technologies) and murine IL-2 (30 U/mL; R&D Systems). Following incubation for 48 hrs at 37°C in the presence of 5% CO_2_, the activated splenocytes were isolated from the Dynabeads using a magnetic sorter and resuspended in FACS buffer at a concentration of 1.5×10^6^ cells/mL. The 100 µL aliquots of splenocyte suspension were transferred into a 96-well V-shaped plate and incubated with antibodies. Activated T cells were stained for flow cytometry with Allophycocyanin (APC)/Cy7-conjugated anti-mouse CD25 antibody (dilution of 1∶50; clone PC61; BD Biosciences). Expression of mouse CCR4 on splenocytes was confirmed using PE-conjugated hamster anti-mouse CCR4 control antibody 2G12 (BioLegend), which was titrated over eight points in a two-fold dilution series, starting at 40 µg/mL (∼267 nM). Biotinylated anti-human CCR4 IgG1 antibodies were titrated the same way and compared with biotinylated anti-human CCR4 antibody 1G1 (BD Biosciences) as well as to a biotinylated isotype control antibody. The bound antibodies were detected using PE-conjugated streptavidin. For evaluation, the PE-signal in the APC/Cy7 anti-CD25 positive population was analyzed, as it should represent the activated T-cell population. In parallel, non-activated splenocytes were stained similarly to test for specificity of the activation and staining procedure.

### Cell Surface Retention

DT40/CCR4^+^ cells (2×10^6^) were incubated for 30 min at 4°C with 1 µg/mL scFv preparations preliminary cross-linked with PE-labeled anti-cMyc IgG (AbD Serotec). In another set of experiments, the cells (2×10^6^) were incubated with 1 µg/mL of IgG preparations labeled with Alexa Fluor 488 using Zenon human IgG labeling kit (Life Technologies). After incubation, the cells were washed in 10 mL FACS buffer, collected by centrifugation at 500 *g* for 5 min at 4 C and mixed with an excess (100 µg/mL) of the corresponding unlabeled IgGs. The decrease of binding was measured by recording the decaying fluorescence signal in flow cytometry. Irrelevant scFv and anti-cMyc-PE alone were included as negative controls for unspecific binding. The kinetic dissociation constant (*k*_off_) and *t*_1/2_ values for dissociation of protein molecules were deduced from a one-phase exponential decay fit of the experimental data using the software program PRISM (GraphPad).

### Platelet Aggregation Assay

Blood (30 mL) was collected by venipuncture with a 21-gauge butterfly needle from healthy volunteers and treated with citric acid to prevent coagulation. Blood donation was received in written consent and in agreement with the REC (Regional Committees for Medical and Health Research Ethics; Oslo/Norway). Platelet rich plasma was obtained by centrifugation at 185 *g* in plastic tubes at room temperature for 15 min. The platelets were incubated either with IgG (9E10J or an isotype control) at a concentration of 10 µg/mL in FACS buffer or with the CCR4 ligands (CCL17 or CCL22) at a concentration 0.25 µg/mL. Adenosine diphosphate (ADP) at a final concentration 3 µM was used as a positive control. Platelet-dependent thrombus formation was detected at 37°C in light transmission aggregometry using a Packs-4 aggregometer (Helena Biosciences, Gateshead, UK) with stirring at 900 rpm. The signal obtained with ADP was set to 100% and the baseline was defined by the platelet depleted serum, which was measured in parallel in all experiments. In parallel, platelet binding measurements were performed by flow cytometry.

### Calcium Flux Assay

The CCRF-CEM target cells, cultivated under regular conditions, were harvested by centrifugation, washed and re-suspended in RPMI-1640 culture medium. One mL containing 2.5×10^6^ cells was mixed with Fluo-4-AM, Pluronic F-127 and Probenecid (all from Life Technologies) to final concentrations of 1 µM, 0.02% and 1 mM, respectively. The cells were incubated at 37°C for 30 min on a vertical rotating wheel (7 rpm). All subsequent steps were carried out in the presence of 1 mM Probenecid. The cells were washed twice in RPMI-1640/10% FBS, once in the assay buffer (145 mM NaCl, 4 mM KCl, 1 mM NaH_2_PO_4_, 0.8 mM MgCl_2_, 25 mM Hepes, 22 mM glucose), and then re-suspended in the assay buffer to a final density of 1.2×10^6^ cells/mL. Aliquots of the cell suspension were mixed with the antibodies and ligands (CCL17, CCL22). The first two components (cells and antibodies) were pre-incubated for 15 min prior to adding the ligand. The final concentrations were 10 µg/mL (67 nM), 10 ng/mL (1.25 nM) and 2.5 ng/mL (0.3 nM) for IgG, CCL17 and CCL22, respectively. As a negative control, an irrelevant chemokine, stromal cell-derived factor-1α (SDF-1α/CXCL12), specific for CXCR4 receptor was used at a concentration of 2.5 ng/mL (0.3 nM). The samples were immediately analyzed using the 515–545 nm band pass filter on a FACSCanto II flow cytometer (BD Biosciences) or on a PHERAstar FS high-throughput microplate reader (BMG Labtech, Offenburg, Germany). Fluorescence was recorded during the time course of 2 min; the areas under the curves (AUC) were integrated using the software PRISM (GraphPad) and plotted as percentage of AUC for maximal stimulation with the ligand alone against the antibody concentrations. The *IC*_50_ values were deduced from the non-linear regression curve fit using a model ‘log [inhibitor] vs. response’ of the software program PRISM (GraphPad).

### Chemotaxis Assay

Chemotaxis experiments were performed in RPMI-1640 medium supplemented with 1% FBS using either ChemoTx (Neuro Probe, Gaithersburg, MD) or Multiscreen-MIC (5 µm pore size; Millipore, Billerica, MA) 96-well plates. The CCR4^+^ CCRF-CEM target cells were loaded in the upper chamber of the transwell plate and chemotaxis was performed in the presence of CCR4 ligands CCL17 or CCL22 at a concentration 3.5 nM (28 ng/mL) in the lower compartment. For characterization of the antagonistic properties, the cells were pre-incubated with IgG1 antibodies in the upper compartment. The chemotaxis plates were incubated at 37°C, 100% humidity, and 5% CO_2_ for 3 hrs. The number of cells migrating into each lower compartment was quantified by flow cytometry using FACSCanto II (BD Biosciences). The number of migrated cells in the presence of the ligand alone was set to 100% migration and the numbers of migrated cells in the presence of ligands and antibodies were expressed as the corresponding percentage. The results were analyzed by fitting the experimental curves (% migrated cells vs. antibody concentration) by non-linear regression using a model ‘log [inhibitor] vs. response’ of the software program PRISM (GraphPad) and the corresponding *IC*_50_ values were calculated.

### Antibody-dependent Cellular Cytotoxicity Assay (ADCC)

The target cells (CCRF-CEM, HUT78 or L428), cultivated under standard conditions, were collected by centrifugation, washed twice and re-suspended in RPMI-1640 medium. One mL containing 2.5×10^6^ cells was mixed with calcein-AM (Life Technologies) to a final concentration of 10 µM and incubated at 37°C for 30 min. The cells were washed three times in RPMI-1640/10% FBS and the cell density was adjusted to 3×10^5^ cells/mL. Human peripheral blood mononuclear cells (PBMC) were prepared from fresh donor blood by Ficoll-Hypaque gradient centrifugation, washed in RPMI-1640/10% FBS and re-suspended at a density 6×10^6^ cells/mL. Fifty µL of target and effector cells were mixed in the same wells of a 96-well microtiter plate thus providing an effector-to-target (E:T) cell ratio of 20∶1. The antibodies were added to the same wells and the plate was incubated at 37°C for 4 hrs. After 3 hrs and 45 min incubation, 20 µL 0.9% Triton X-100 was added to the control wells to achieve complete lysis of the target cells (referred as maximal lysis). One hundred µL supernatant of each sample was then transferred into a black microtiter plate and the fluorescence (excitation at 488 nm, emission at 518 nm) was recorded using a Tecan M200 plate reader. Each experiment was carried out in quadruplicate. The fluorescence intensity of the samples without antibodies was subtracted as a background and the percentage of specific lysis in samples with antibodies was calculated. To determine *EC*_50_ values (effective concentrations leading to 50% maximal killing), the dose-response curves were computed by a nonlinear regression analysis and a three-parameter fit model ‘log [agonist] vs. response’ using the software program PRISM (GraphPad). Blood samples were supplied as buffy coats from the bloodbank of the University Hospital of Oslo under license and in accordance with Norwegian Law Isolation (Regional Committees for Medical and Health Research Ethics; Oslo/Norway).

### Isolation and Staining of T_reg_ Cells

Human T_reg_ cells were isolated from buffy coats using a Dynabeads Regulatory CD4^+^ CD25^+^ T cell kit (Life Sciences) according to the protocol of the manufacturer. Upon harvesting from PBMC, the T_reg_ cells were collected by centrifugation and re-suspended in FACS buffer to a cell density of 10^6^ cells/mL. Two hundred µL with 2×10^5^ PBMC were mixed with 100 µL suspension of T_reg_ cells and transferred into a 96 well V-shaped plate followed by centrifugation at 400 *g* for 5 min at 4°C. The cell pellets were resuspended in 50 µL FACS buffer, mixed with the same volumes of biotinylated anti-CD127 or anti-CCR4 antibody (both from BD Biosciences) and incubated at 4°C for 1 hr. The samples were washed three times with FACS buffer and collected by centrifugation as described above. The cell pellets were resuspended in either 50 µL of FACS buffer alone or in the same buffer containing anti-CD4-FITC (eBioscience, Hatfield, UK), anti-CD25-APC (eBioscience), Streptavidin-PerCP (BD Biosciences) with and without anti-CCR4-PE (BD Biosciences) followed by incubation at 4°C for 1 hr. The samples were washed three times with FACS buffer, and the cells were collected by centrifugation as described above. The cell pellets with anti-CCR4-PE and unstained were resuspended in 200 µL of FACS buffer and transferred into a 96-well U-shaped plate for analysis by flow cytometry using the FACSCantoII flow cytometer (BD Biosciences). The remaining samples were stained with anti-FoxP3 antibody (BD Biosciences) according to the protocol described in the FoxP3 Staining Buffer Set (eBiosciences). At the end of the staining procedure, the cells were resuspended in 200 µL of FACS buffer and transferred into a 96-well U-shaped plate for analysis by flow cytometry using FACSCantoII (BD Biosciences). The ADCC experiments using isolated T_reg_ cells were performed as described above, except that the E:T ratio was lowered to 15∶1 and the antibodies were used at a single concentration of 3.3 nM in triplicates. To check whether the PBMCs were functional, the ADCC experiment was performed in parallel on CCRF-CEM target cells. Blood samples were supplied as buffy coats from the bloodbank of the University Hospital of Oslo under license and in accordance with Norwegian Law Isolation (Regional Committees for Medical and Health Research Ethics; Oslo/Norway).

### Antibody-dependent Cellular Phagocytosis Assay (ADCP)

Human monocytes were isolated from buffy coats following the RosetteSep procedure (Stemcell Technologies, Grenoble, France) and stained for 30 min on ice with APC-conjugated anti-CD14 antibody (BD Biosciences). Stained monocytes were washed in FACS buffer and collected by centrifugation at 300 *g* for 5 min at 4°C. A cell aliquot was used to count the live and dead cells. In parallel, 3.0×10^6^ target cells (CCRF-CEM or 786-O) were labeled using 17 µL of carboxyfluorescein succinimidyl ester (CFSE) stock solution (260 µM; Life Technologies) for 15 min at 37°C. The labeling reaction was stopped by addition of RPMI-1640/10% FBS and the cells were collected by centrifugation at 300 *g* for 4 min at 4°C. The labeled cells were resuspended in fresh RPMI-1640/10% FBS. The isolated effector cells (monocytes) were mixed with the labeled target cells (CCRF-CEM or 786-O) at E:T ratio of 5∶1 and loaded into a V-shaped 96-well plate. Different antibody dilutions were added to the wells and the plate was incubated at 37°C for 1, 2 or 3 hrs. The reaction was stopped by addition of 50 µL of 2% formalin. The plates were analyzed by flow cytometry on a FACSCantoII flow cytometer (BD Biosciences) using the FITC- and APC-channels, for detection of CFSE-labeled target cells and monocytes, respectively. Phagocytosis was calculated as a ratio of the number of double positive cells appearing in the FITC/APC-gate to the number of CFSE-events (labeled target cells) and expressed in percentage. Blood samples were supplied as buffy coats from the bloodbank of the University Hospital of Oslo under license and in accordance with Norwegian Law Isolation (Regional Committees for Medical and Health Research Ethics; Oslo/Norway).

### Human T-cell Lymphoma Xenograft Model

Male NMRI:nu/nu mice were purchased from Taconic Europe (Lille Skensved, Denmark). The animals were housed under pathogen-free conditions in individually ventilated cages under standardized environmental conditions (22°C room temperature, 50±10% relative humidity, 12 hours light-dark rhythm). They received autoclaved food and bedding (Ssniff, Soest, Germany) and acidified (pH 4.0) drinking water ad libitum. Ten mice per group, at an age of 6–8 weeks, were subcutaneously (s.c.) injected with CCRF-CEM cells (2×10^7^). Animals were randomized into treatment groups approximately three weeks after tumor inoculation (TI) when the tumors reached the size of 60–100 mm^3^; 5–8 mice per group with equal tumor size were selected for the treatment. The antibodies were injected intravenously (i.v.) at a dose of 10 mg/kg twice a week for three weeks. The control groups were treated with PBS. Tumor size was measured in two dimensions with a caliper-like instrument. Individual tumor volumes (V) were calculated by the formula V = 0.5×(length+width)^2^. Upon reaching the humane endpoint with a tumor volume of 1,500 mm^3^, the animals were sacrificed by cervical dislocation. The Kaplan-Meier survival plots were generated using the software program PRISM (GraphPad) and the survival curves were compared using a log-rank (Mantel-Cox) test. All animal experiments were performed in compliance with national laws and guidelines, i.e. according to the German Animal Protection Law and with approval from the responsible authorities (LAGeSo, State Office of Health and Social Affairs; Berlin, Germany). The *in vivo* procedures were consistent and in compliance with the UKCCCR guidelines.

## Results

### Generation of Anti-CCR4 Antibodies *In Vitro* by Cell-based Antibody Selection

**Figure 1:**
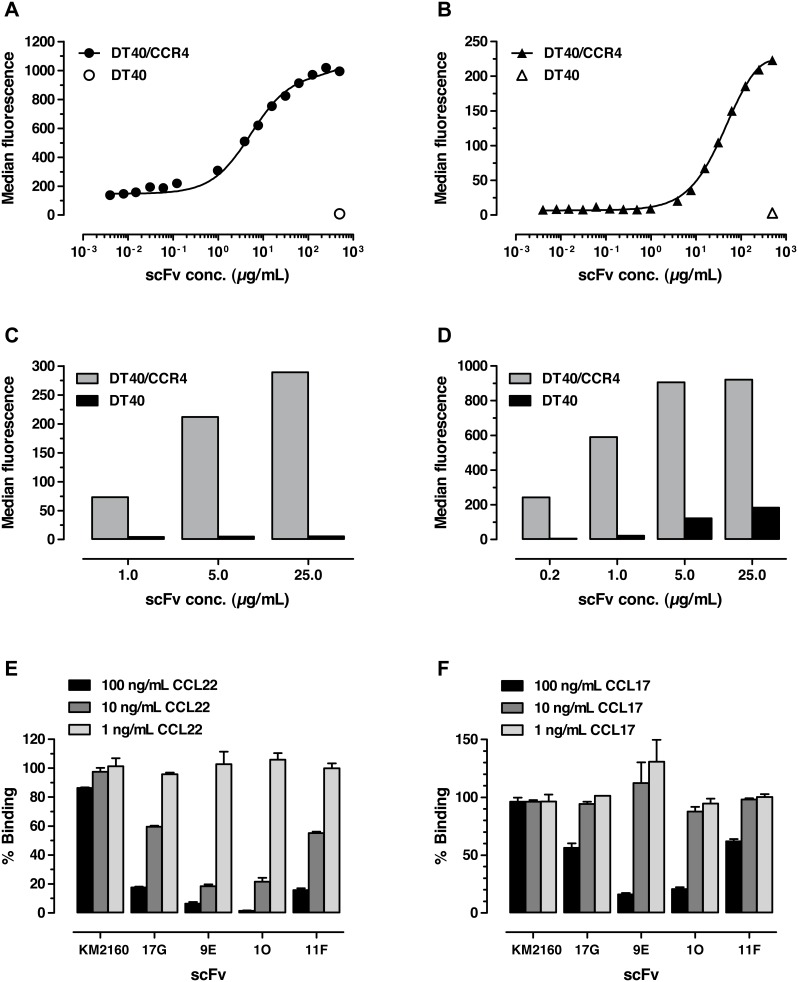
Binding of anti-CCR4 scFv fragments to avian DT40 cells transfected with human CCR4. The results of titration on DT40/CCR4 and non-transfected DT40 cells are shown for scFv 17G (**a**), 9E (**b**), 1O (**c**) and 11F (**d**). Binding of scFv fragments in the presence of increasing concentrations of CCR4 ligands CCL22 and CCL17 is shown in panels (**e**) and (**f**), respectively. As a control, a scFv fragment derived from hybridoma KM2160 was used. Cell binding was analyzed by flow cytometry; bound scFv fragments were detected with anti-cMyc antibody followed by anti-human-PE immunoconjugates. Median fluorescence intensity is plotted against the scFv concentration (µg/mL); the results of representative experiments from three repeats are shown.

In order to generate initial antibodies against CCR4, phage display selection was performed using a scFv antibody library derived from a naïve human IgM/IgD repertoire [33]. To identify antagonistic antibodies against CCR4, the phages binding to the CCR4^+^ cells and not to CCR4^−^ cells were competitively eluted in presence of CCR4-specific ligands CCL17 or CCL22. The second strategy included elution using trypsin or citric acid to generate antibodies that could potentially have either higher affinities than the CCR4 ligands, CCL17 and CCL22, or bound to epitopes outside of the ligand-binding pocket on the receptor. After completion of three rounds of panning, the selected phage pools were subjected to screening in cell binding assays using a cell reporter-assisted high-throughput screening system. Screening of 12,240 clones resulted in identification of 132 scFv candidates binding CCR4^+^ cells and showing no binding to CCR4^−^ cells. Sequencing led to identification of four different CCR4-specific antibody variants, 17G, 9E, 1O and 11F. These scFv fragments demonstrated specific binding to cells transfected with the gene encoding human CCR4 (Figure 1a–d) and to cell lines naturally expressing CCR4 (not shown). In contrast, none of the four selected candidates showed significant binding to CCR4-negative cells (Figure 1a–d). Analysis of CCR4^+^ cell binding in presence of increasing concentrations of CCR4 ligands CCL22 and CCL17 demonstrated a clear decrease in staining signals for all scFv variants with both CCL22 and CCL17 (Figure 1e, f). In contrast, no effect of CCL17 or CCL22 was observed on binding of a control scFv fragment derived from the murine hybridoma KM2160 recognizing the N-terminal part of human CCR4 [30]. The results indicated that the antibodies 17G, 9E, 1O and 11F interfered with ligand binding and thus might have CCR4-blocking activity.

### *In vitro* Characterization of the Selected Human Anti-CCR4 Antibodies

**Figure 2:**
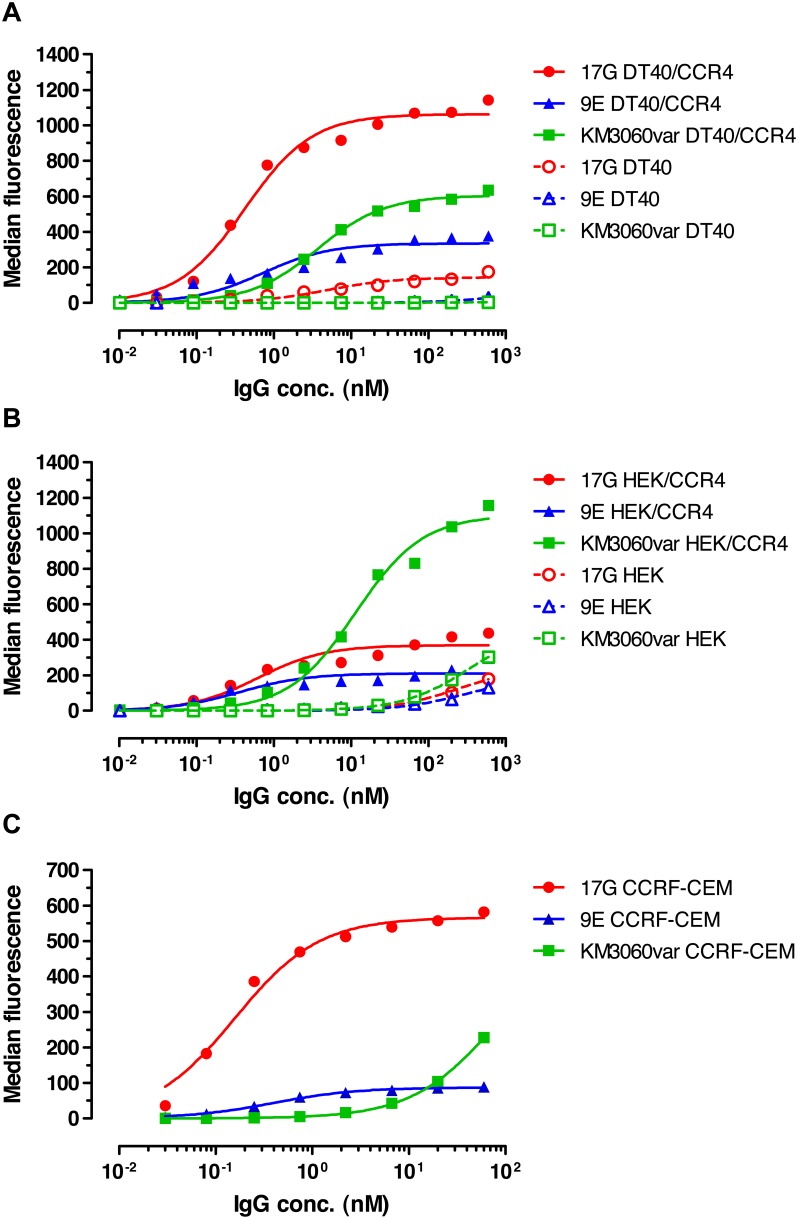
Dose-dependent binding of anti-CCR4 IgG antibodies to CCR4^+^ and CCR4^−^ cell lines. Different concentrations of 17G, 9E and KM3060var antibodies were tested in flow cytometry for binding to avian DT40 cells stably transfected with human CCR4 (**a**), to human HEK-293 cells transiently transfected with human CCR4 (**b**) or to human T-cell leukemia line CCRF-CEM naturally expressing CCR4 (**c**). Binding to CCR4-negative non-transfected cells is also shown in panels (**a**) and (**b**). The results of representative experiments from three repeats are shown.

The selected scFv candidates 17G and 9E were reformatted as full length human IgG1 for further characterization. The variant of the KM2160-derived chimeric (mouse variable domains/human constant domains) IgG1 antibody KM3060 [30] made in-house (KM3060var) was used as a positive control. The anti-CCR4 IgG antibodies 17G, 9E and KM3060var demonstrated specific binding to an avian cell line stably transfected with human CCR4 gene (DT40, receptor density ∼92,000 CCR4 molecules per cell), to a human cell line transiently transfected with human CCR4 gene (HEK-293, receptor density ∼46,000 CCR4 molecules per cell) and to a human T-lymphoblastic leukemia cell line that endogenously expresses CCR4 (CCRF-CEM, receptor density ∼6,200 CCR4 molecules per cell), as determined by flow cytometry (Figure 2). No significant binding was observed to the tested non-transfected host cells.

**Figure 3:**
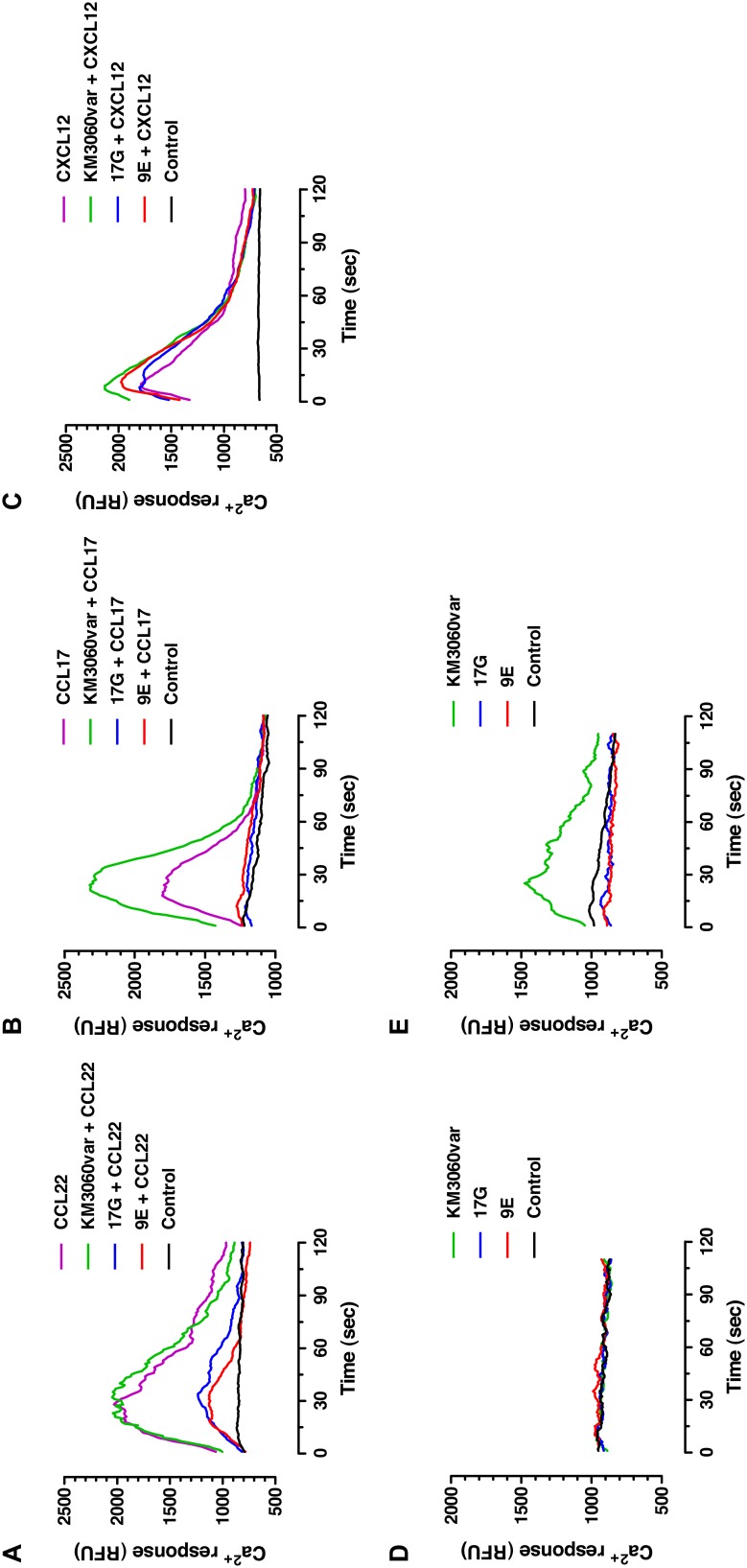
Effect of antibodies on ligand-induced signaling using calcium flux assay. CCR4-positive CCRF-CEM cells loaded with the Ca^2+^ sensing dye Fluo-4 were pre-incubated with 10 µg/mL of IgGs KM3060var, 17G or 9E for 15 min before adding CCR4-specific ligands, CCL17 or CCL22 at final concentrations of 10 ng/mL and 2.5 ng/mL, respectively. As a negative control, an irrelevant chemokine, stromal cell-derived factor-1α (SDF-1α/CXCL12), specific for CXCR4 receptor was used at a concentration of 2.5 ng/mL. Time course data from calcium flux assays were expressed in terms of relative fluorescent units (RFU). The black line in all panels represents the signal from the non-treated cells (control). Effect of antibodies on cell signaling induced by the ligands CCL22, CCL17 and CXCL12 is shown in panels (**a**), (**b**) and (**c**), respectively. Effect of antibodies alone at concentrations 10 µg/mL and 100 µg/mL on cell signaling is shown in panels (**d**) and (**e**), respectively.

The IgG candidates 17G and 9E interfered with the ligand-induced cell signaling, as demonstrated by inhibition of calcium mobilization (Figure 3, Table 1). In contrast, no inhibitory effect was observed for the antibody KM3060var used as a control. Interestingly, the antibody KM3060var demonstrated a slight agonistic activity when used at high concentrations (Figure 3e).

**Table 1:** Effect of anti-CCR4 antibodies on calcium mobilization induced by CCR4-specific ligands CCL22 and CCL17 and by irrelevant ligand CXCL12.

Antibody	Chemokine		
	CCL22	CCL17	CXCL12
**KM3060var**	100.98	220.41	115.42
**17G**	25.70	0.00	103.78
**9E**	11.95	0.00	107.38

CCR4-positive CCRF-CEM cells, loaded with the Ca^2+^ sensing dye Fluo-4, were stimulated with either CCR4-specific ligands CCL22 and CCL17 or the irrelevant chemokine CXCL12, in presence or absence of antibodies. Samples were analyzed by FACS and the AUC value, corresponding to CCR4 receptor activation, was recorded over 2 min. Ratios of integrated AUCs expressed as percentage of AUC for stimulation with the ligand alone are shown.

**Figure 4:**
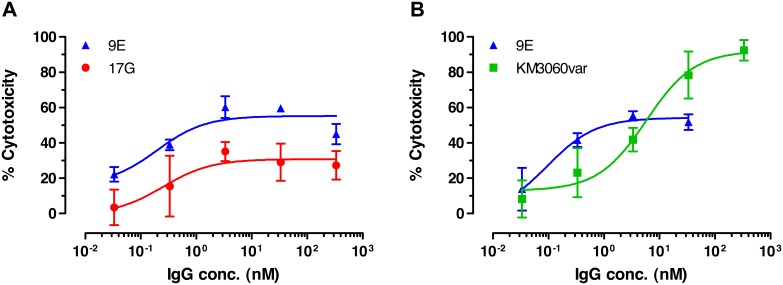
ADCC activity of anti-CCR4 antibodies. Representative experiments demonstrating dose dependent killing of CCRF-CEM cells is shown. (**a**) Comparison of ADCC activity of human IgG1 variants 17G and 9E. (**b**) Comparison of killing activity of the human IgG1 variant 9E with a comparator antibody KM3060var. Mean and SD values of quadruplicates are plotted.

Both human antibody variants 9E and 17G possessed strong ADCC activity against CCR4^+^ cells, with higher maximal killing demonstrated by the antibody 9E (Figure 4; Table 2). Due to its superiority in signal inhibition and ADCC activity in comparison to other selected anti-CCR4 antibodies, the variant 9E was selected for further improvement by affinity maturation.

**Table 2:** Comparative ADCC activity of anti-CCR4 antibodies.

	9E	17G	KM3060var
**Max killing (%)**	42.7±12.7	28.1±6.5	70.0±15.5
***EC*** **_50_ (ng/mL)**	256.2±123.3	45.0±20.7	335.0±70.0
**N**	8	7	11

CCR4-positive CCRF-CEM cells were labeled with calcein and incubated with isolated human PBMCs as effector cells in presence of different concentrations of anti-CCR4 antibodies. Induction of ADCC was expressed as percentage of cells lysed; the maximal lysis using detergent was set to 100%. Means and SDs from 7–11 independent experiments are shown.

### Affinity Maturation of Anti-CCR4 Antibody 9E and Cell-binding properties of the Derived Variants

**Figure 5:**
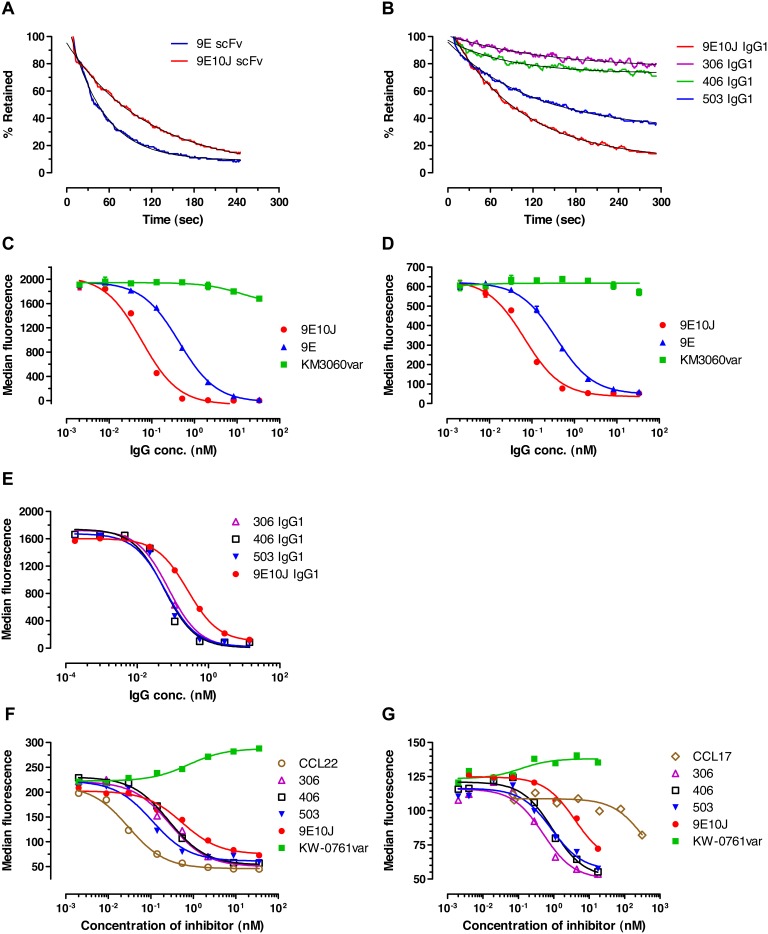
Affinity assessment of the improved anti-CCR4 antibodies. (**a**, **b**) Analysis of dissociation rates in the cell surface retention assay *in vitro* on DT40/CCR^+^ cells. Either scFv molecules cross-linked with PE-labeled anti-cMyc antibody (**a**) or human IgG1 molecules labeled with Alexa Fluor 488 (**b**) were used for cell binding. In all experiments the unlabeled 9E10J IgG1 molecules were added to the cells to prevent rebinding of labeled molecules; decay of the fluorescence signal was recorded. Values are expressed as a percentage of the initial median fluorescence intensity. (**c–e**) Inhibition of biotinylated antibodies 9E (**c**, **d**) and 9E10J (**e**) binding to DT40/CCR4 (**c**, **e**) or CCRF-CEM cells (**d**) in the presence of increasing concentrations of unlabeled IgGs 9E, 9E10J, 306, 406, 503 and control antibody KM3060var. (**f, g**) Inhibition of labeled ligands, CCL22 (**f**) or CCL17 (**g**), binding to DT40/CCR4 cells in the presence of increasing concentrations of unlabeled IgG 9E10J, 306, 406, 503, control antibody KW-0761var and of the corresponding unlabeled ligand.

Affinity maturation was performed using the previously described chain shuffling and site-directed mutagenesis approaches [41]. The resulting variant 9E10J and its derivatives, such as antibodies 306, 406 and 503, were used for more detailed characterizations. Direct cell binding and cell surface retention experiments demonstrated that the variant 9E10J had 3–4-fold affinity enhancement towards human CCR4 in comparison with the parental variant 9E (Figure 5a; Tables 3, 4). The mutated variants 306, 406 and 503 additionally had more than ten-fold improved binding affinity towards the CCR4-positive cells in comparison to the antibody 9E10J (Figure 5b; Tables 3, 4). The competitive inhibition experiments demonstrated that the antibodies 9E, 9E10J, 306, 406 and 503 recognized substantially the same epitope on CCR4 receptor and could inhibit binding of each other but not of the control antibody KM3060var (data not shown). The *IC*_50_ values derived from the inhibition experiments demonstrated 5–7-fold affinity improvement of the variant 9E10J over the antibody 9E (Figure 5c, d; Table S1a in File S1) and further 3–4-fold affinity enhancement for the antibodies 306, 406 and 503 (Figure 5e; Table S1b in File S1). In addition, the 9E-derived antibody variants effectively inhibited binding of labeled CCR4 ligands, CCL17 and CCL22, to the receptor, with lowest *IC*_50_ values demonstrated by the affinity matured antibodies 306 and 503 (Figure 5f, g; Table S1c in File S1). As expected, the control antibodies KM3060var and KW-0761var (both recognize the same epitope in N-terminal part of CCR4) did not inhibit binding of human anti-CCR4 antibodies generated in this study and the CCR4-specific ligands (Figure 5c, d, f, g).

**Table 3:** Apparent affinities of anti-CCR4 antibodies (human IgG1 format) as determined in cell binding experiments.

Cell line	Affinity (*K*_D_, nM) of antibody				
	9E	9E10J	306	406	503
**DT40/CCR4**	4.95±2.67	1.17±0.45	0.03±0.00	0.15±0.02	0.04±0.01
**CCRF-CEM**	0.37±0.11	0.13±0.03	0.052±0.09	0.18±0.04	0.02±0.00
**HUT78**	n.d.	**-**	0.02±0.00	0.09±0.02	0.05±0.01
**L428**	n.d.	**-**	9.63±4.94	2.97±1.48	11.22±8.36

Binding of diluted anti-CCR4 antibodies to different cell lines was measured using flow cytometry. Recorded median fluorescence intensity values were plotted against antibody concentrations and the affinities were calculated using the ‘one-site-total binding’ equation of the software PRISM (GraphPad). n.d., not determined; **-**, not detectable.

**Table 4:** Cell surface retention of anti-CCR4 antibodies.

Molecule	Off-rate (1/s)	*t*_1/2_ (min)
scFv 9E	2.10×10^−2^	0.55
scFv 9E10J	8.78×10^−3^	1.32
IgG1 9E10J	7.6×10^−3^	1.50
IgG1 306	7.5×10^−4^	15.00
IgG1 406	8.3×10^−4^	14.00
IgG1 503	3.5×10^−3^	3.50

Labeled anti-CCR4 antibodies were incubated with CCR4-positive DT40-cells and the decrease of fluorescent signals during the time course in the presence of excessive amounts of the corresponding unlabeled antibodies were recorded using flow cytometry. To prevent rebinding of labeled molecules, the unlabeled 9E10J IgG1 was added to the cells in all experiments. The off-rate (*k*_off_) and half-life (*t*_1/2_) values for dissociation of the antibody-antigen complexes were interpolated from the standard curves generated by one-phase exponential decay fit of the experimental data as shown in Figure 5a, b.

**Figure 6:**
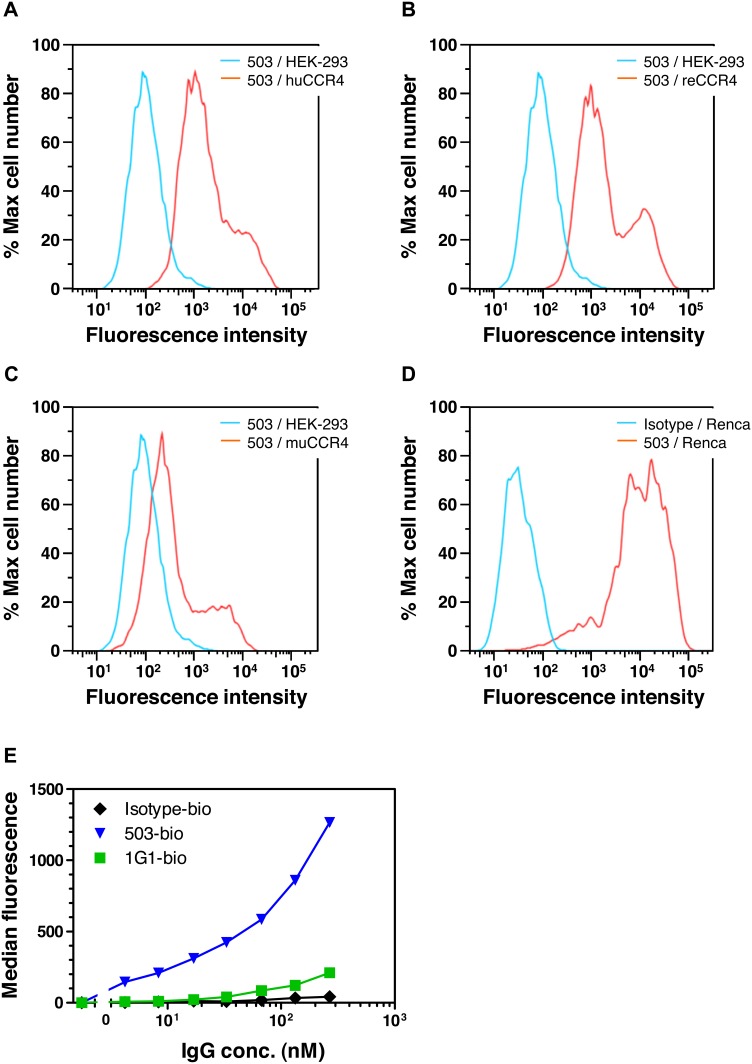
Analysis of species cross-reactivity in cell-binding experiments. (**a–c**) Human IgG1 antibody 503 (1 µg/mL) demonstrates binding to HEK-293 cells transiently expressing either human CCR4 (huCCR4; **a**) or the receptor from rhesus macaque (reCCR4; **b**), or from mouse (muCCR4; **c)**. As negative controls, binding of the antibody 503 to mock-transfected HEK-293 cells is shown. (**d**) Human IgG1 antibody 503 (2 µg/mL) specifically binds to the mouse kidney cancer cell line Renca. (**e**) Dose-dependent binding of biotinylated IgG1 503 to activated mouse splenocytes. In addition, binding patterns of the isotype control and anti-human CCR4 commercial antibody 1G1 are shown.

To test whether the generated antibodies cross-react with CCR4 receptor of other species, we analyzed antibody binding to HEK-293 cells transiently transfected with genes encoding CCR4 from mouse and rhesus macaque (*Macaca mulatta*). The HEK-293 cells transfected with the human CCR4 were used as a positive control for binding. The results demonstrated specific interaction of the human antibodies to CCR4 from man, non-human primates and mouse (Figure 6a–c), with similar binding patterns for all the affinity improved variants 306, 406 and 503 (data not shown). Binding to murine CCR4 was additionally confirmed in the flow cytometry experiments using CCR4^+^ murine renal cell carcinoma cell line Renca (Figure 6d) and the mouse splenocytes, which express CCR4 upon activation via CD3 and CD28 [42] (Figure 6e). The lower binding signals detected for antibody binding to murine CCR4 (Figure 6c) could be caused either by a difference in expression levels of the antigen on the HEK-293 cells and/or by a difference in the antibody affinity towards the antigen from different species. Regardless of potential differences in affinity, the data clearly indicate that the antibodies cross-react with CCR4 receptors from different species.

### Effect of Human Anti-CCR4 Antibodies on Platelet Aggregation

**Figure 7:**
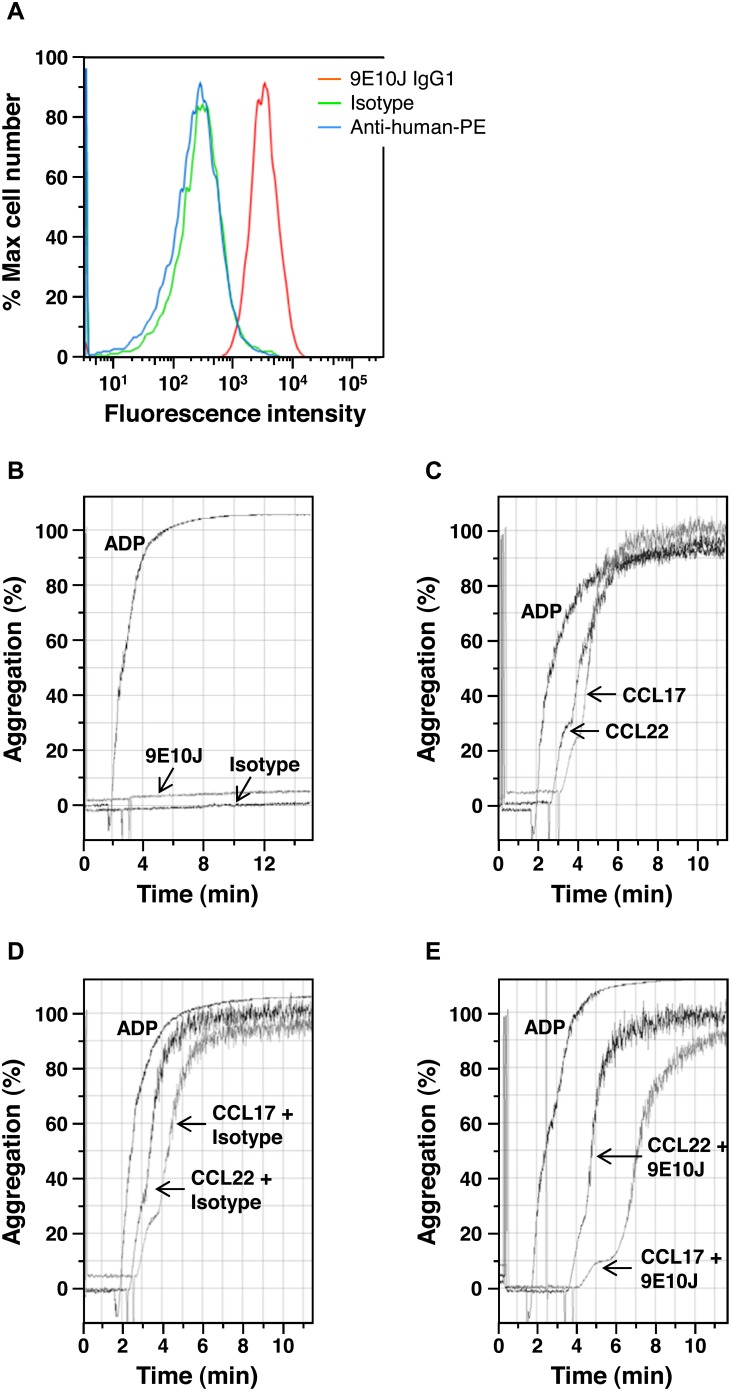
Effect of human anti-CCR4 antibody 9E10J on platelet aggregation. (**a**) Binding of 9E10J IgG1 at concentration 10 µg/mL to human platelets isolated from the fresh donor blood. As negative controls, binding histograms for an isotype control antibody (Isotype) and of a secondary antibody alone (anti-human-PE) are shown. (**b**) Effect of anti-CCR4 antibody 9E10 and of the isotype control antibody on platelet aggregation in comparison with ADP. (**c**) Platelet aggregation induced by CCR4 ligands, CCL17 and CCL22, in comparison with ADP. (**d, e**) Ligand-induced aggregation of platelets pre-incubated with either the isotype control antibody (**d**) or anti-CCR4 antibody 9E10J (**e**).

Expression of functional CCR4 on human platelets [43] raised the question whether the isolated human antibodies would bind to platelets and affect their activation. Experiments performed with an affinity-matured variant 9E10J demonstrated that the antibody indeed binds to platelets (Figure 7a), however, this binding did not lead to platelet activation nor aggregation (Figure 7b). In contrast, the CCR4 ligands when incubated with platelets strongly induced their aggregation (Figure 7c). This effect of ligands was not changed in presence of the isotype control antibody (Figure 7d) and was only slightly influenced by the anti-CCR4 antibody 9E10J at a concentration used in the assay (Figure 7e).

### Inhibition of CCR4-mediated Signaling and Chemotaxis

**Figure 8:**
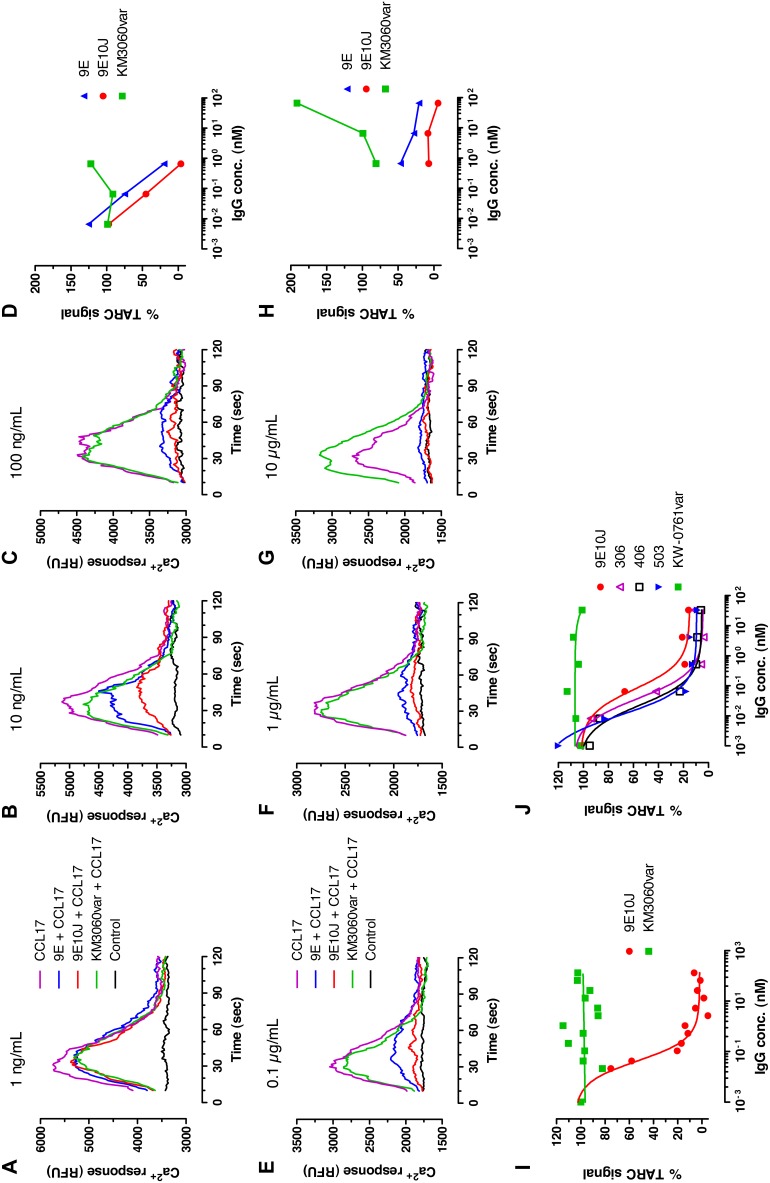
Dose-dependent effect of anti-CCR4 antibodies on CCL17-induced signaling using calcium flux assay. The results of four independent experiments on CCRF-CEM cells loaded with Fluo-4 using different concentration intervals are shown in panels (**a–d**), (**e–h**), (**i**) and (**j**). The cells were pre-incubated with human IgG1 variants 9E, 9E10J, 306, 406, 503 or with the control antibodies KM3060var and KW-0761var for 15 min before adding a CCR4-specific ligand CCL17 at a concentration of 10 ng/mL. The IgG concentrations were either 1 (**a**), 10 (**b**) and 100 ng/mL (**c**) or 0.1 (**e**), 1.0 (**f**) and 10 µg/mL (**g**) in the first and second set of experiments, respectively. The areas under the curves (AUC) were integrated using software PRISM (GraphPad) and plotted as percentage of AUC for maximal stimulation with CCL17 alone against antibody concentrations, as shown in panels (**d**) and (**h**) for the first and second set of experiments, respectively. The results of other two independent experiments using broader range of antibody concentrations are shown in panels (**i**) and (**j**).

For functional characterization of the affinity improved human anti-CCR4 antibodies, their ability to inhibit CCR4-mediated intracellular signaling was measured. As shown in Figure 8 and Table S2 in File S1, the affinity improved human anti-CCR4 antibodies demonstrated dose-dependent inhibition of the CCL17-induced signaling in a calcium-flux assay, with five-fold higher potency demonstrated by 9E10J variant over the parental antibody 9E. The signal induced by CCL17 at a concentration of 1.25 nM was completely inhibited by the antibody 9E10J at the concentrations of 0.7–2.0 nM (Figure 8i). The mutated antibodies 306, 406 and 503 showed a 2-6-fold further improvement in potency (Figure 8j; Table S2b in File S1). These variants also demonstrated strong inhibition of signaling induced by the high affinity CCR4 ligand CCL22 (Table S2c in File S1). As expected, the comparator antibodies KM3060var and KW-0761var did not show any antagonistic activity (Figure 8). Moreover, the control antibody KM3060var revealed receptor agonistic activity in some experiments when used at high concentrations (Figure 8g, h).

**Figure 9:**
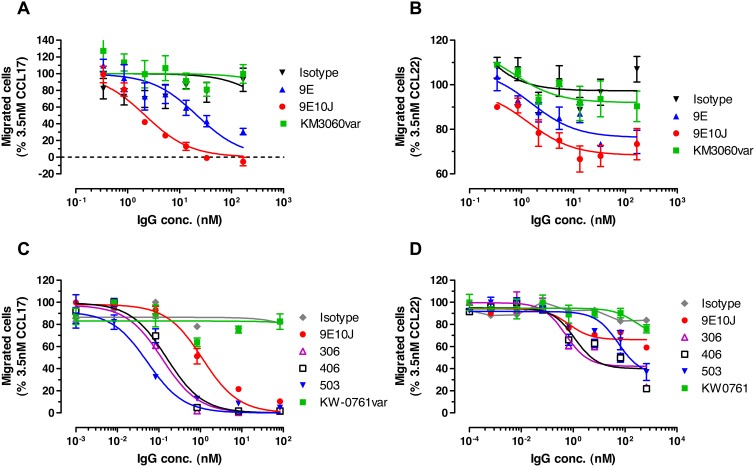
Dose-dependent effect of anti-CCR4 antibodies on CCL17 or CCL22-induced chemotaxis of CCR4^+^ CCRF-CEM cells. Human T-cell leukemia CCRF-CEM cells were induced to migrate in the transwell plates, where the CCR4 ligands were placed in the lower chamber and the cells were co-incubated with either CCR4-specific antibodies or isotype control antibody in the upper chambers. The cells migrated to the lower chamber were detected and counted by flow cytometry. (**a**, **c**) Inhibition of CCL17-induced migration. (**b**, **d**) Inhibition of CCL22-induced migration. Mean and SD values of triplicates are plotted.

The ability of anti-CCR4 human antibodies to inhibit ligand-induced chemotaxis was next investigated to determine whether the observed affinity gain could result in enhanced potency to prevent receptor-mediated cell migration. As shown in Figure 9 and Table S3 in File S1, the human anti-CCR4 antibodies demonstrated dose-dependent inhibition of the ligand-induced cell migration. The affinity improved variant 9E10J demonstrated ten-fold higher potency (lower *IC*_50_ values) than the library-derived variant 9E in inhibiting CCL17-induced chemotaxis (Figure 9a). The mutated variants 306, 406 and 503 showed additional 7-22-fold enhancement of potency in inhibiting CCL17-induced chemotaxis over the antibody 9E10J (Figure 9c). The observed potency enhancement also positively correlated with increased percentage of maximal chemotaxis inhibition. In contrast, less pronounced improvement of *IC*_50_ values was observed for inhibition of chemotaxis induced by the high-affinity CCR4 ligand CCL22, although the affinity improved variants demonstrated significantly better maximum inhibition (Figure 9b, d; Table S3 in File S1). The comparator antibodies KM3060var and KW-0761var did not show any effect on ligand-induced chemotaxis (Figure 9).

### ADCC and ADCP Activities of Affinity Matured Human Anti-CCR4 Antibodies

**Figure 10:**
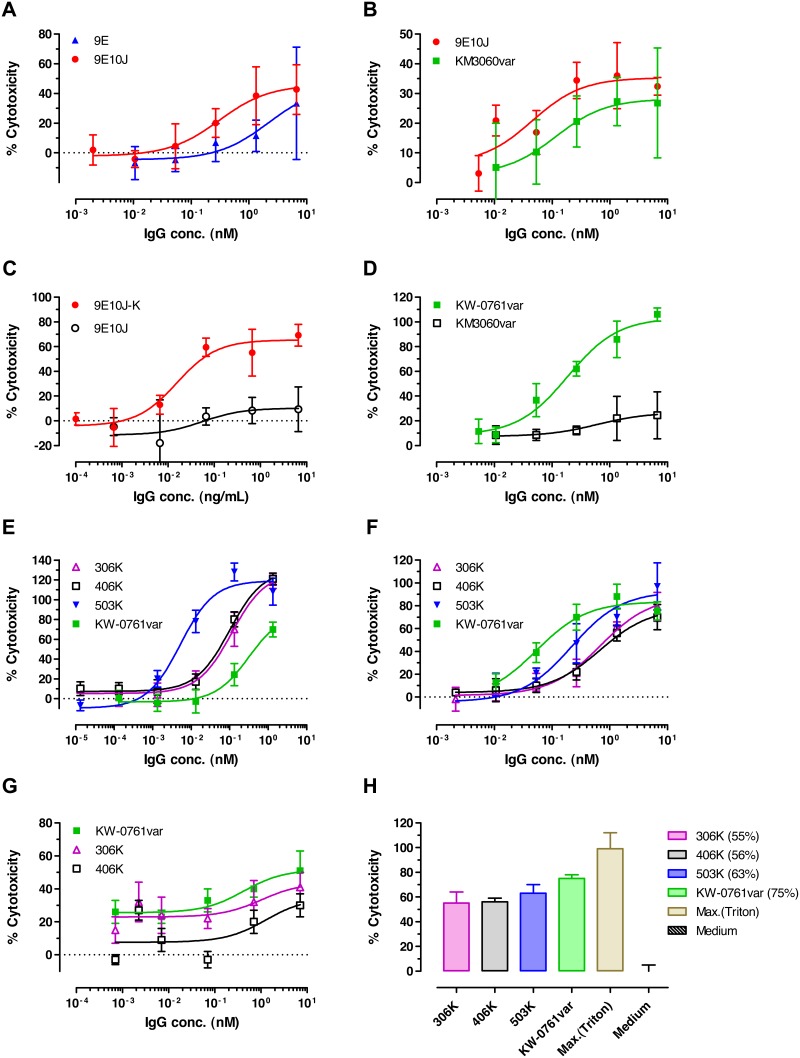
ADCC activity of anti-CCR4 antibodies using different tumor cell lines and autologous T_reg_ cells as targets. (**a**, **b**) Comparison of the ADCC activity on T-cell leukemia CCRF-CEM cells of the affinity improved variant 9E10J to its parent antibody 9E (**a**) and a comparator antibody KM3060var (**b**). (**c**, **d**) Analysis of kifunensine effect (indicated with K) on ADCC activity of the human antibody 9E10J (**c**) and of the comparator antibodies KM3060var (not treated with kifunensine) and defucosylated KW-0761var (**d**) using CCRF-CEM as target cells. (**e–g**) ADCC activity of the defucosylated affinity improved anti-CCR4 antibodies 306 K, 406 K, 503 K (K means kifunensine treatment) and of the comparator antibody KW-0761var, prepared under the same conditions, on CCRF-CEM cells (**e**), Hodgkin’s lymphoma L428 cells (**f**) and cutaneous T-cell lymphoma HUT78 cells (**g**). (**h**) Comparative ADCC activity of anti-CCR4 antibodies on isolated autologous T_reg_ cells. The IgG1 molecules were used at a concentration of 3.5 nM. Cytotoxicity was normalized to a maximum release (100% cell lysis) in presence of Triton X-100. The killing activity (%) is shown in brackets. Typical experiments from 3–8 repeats are shown. Mean and SD values of quadruplicates are plotted.

The affinity matured human anti-CCR4 antibodies were further characterized for their capacity to mediate ADCC activity using human PBMC as effector cells. Head-to-head comparison of the antibody variant 9E10J with its parent clone 9E demonstrated a slight increase in maximum killing and a 7.5-fold decrease in *EC*_50_ value (Figure 10a; Table S4a in File S1). The antibody 9E10J also demonstrated superiority in ADCC activity over the control chimeric antibody KM3060var (Figure 10b; Table S4a in File S1).

To further increase killing capacity of the anti-CCR4 antibodies, the fucose-devoid versions of IgG1 were generated by metabolic modulation of the antibody producing mammalian cells using a mannosidase I inhibitor, kifunensine [39]. Analysis of the antibody glycoforms produced using liquid chromatography–mass spectrometry (LC-MS) demonstrated that 95% of N-linked oligosaccharide structures belonged to a fucose-lacking high-mannose type (data not shown). These antibody species demonstrated significantly increased ADCC effect resulting in much higher levels of cell killing and significantly lower *EC*_50_ values (Figure 10c, d; Table S4b in File S1). Comparison of the affinity matured human antibodies 306, 406 and 503 produced from the mammalian cells treated with kifunensine (K) demonstrated that the antibody variant 503 K had the highest killing capacity towards the T-cell leukemia CCRF-CEM cells with an *EC*_50_ value in the single digit picomolar range (Figure 10e; Table S4c in File S1). The defucosylated human anti-CCR4 antibodies also demonstrated strong ADCC activity against Hodgkin’s lymphoma and cutaneous T-cell lymphoma cell lines (Figure 10f, g, Table S4c in File S1) and against the autologous T_reg_ cells (Figure 10h).

**Figure 11:**
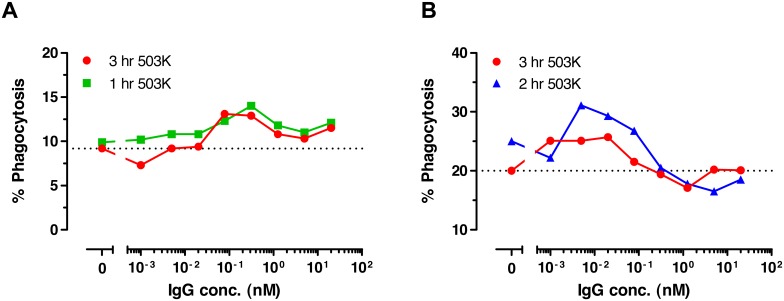
ADCP activity of defucosylated anti-CCR4 IgG1 antibody 503 K in the presence of isolated human monocytes. Dose-dependent phagocytosis of T-cell leukemia CCRF-CEM cells (**a**) and human renal cell cancer 786-O cells (**b**) incubated with monocytes at E:T ratio 5∶1 for 1, 2 or 3 hrs is shown. Dotted lines indicate the levels of spontaneous phagocytosis.

In order to investigate whether the candidate 503 can mediate phagocytic activity of macrophages (ADCP), a phagocytosis assay with human monocytes as effector cells was established. The defucosylated candidate 503 K demonstrated significant enhancement of spontaneous phagocytosis of both T-cell leukemia CCRF-CEM cells and of the human renal cell carcinoma 786-O cells (Figure 11a and b, respectively). Taken together, the affinity matured human anti-CCR4 antibodies, in particular the variant 503, showed improved ADCC and ADCP effector functions, and the ADCC activity could be further enhanced through glycooptimization.

### Anti-tumor Activity of Anti-CCR4 Antibodies in a Xenograft Model of Human T-cell Lymphoma

**Figure 12:**
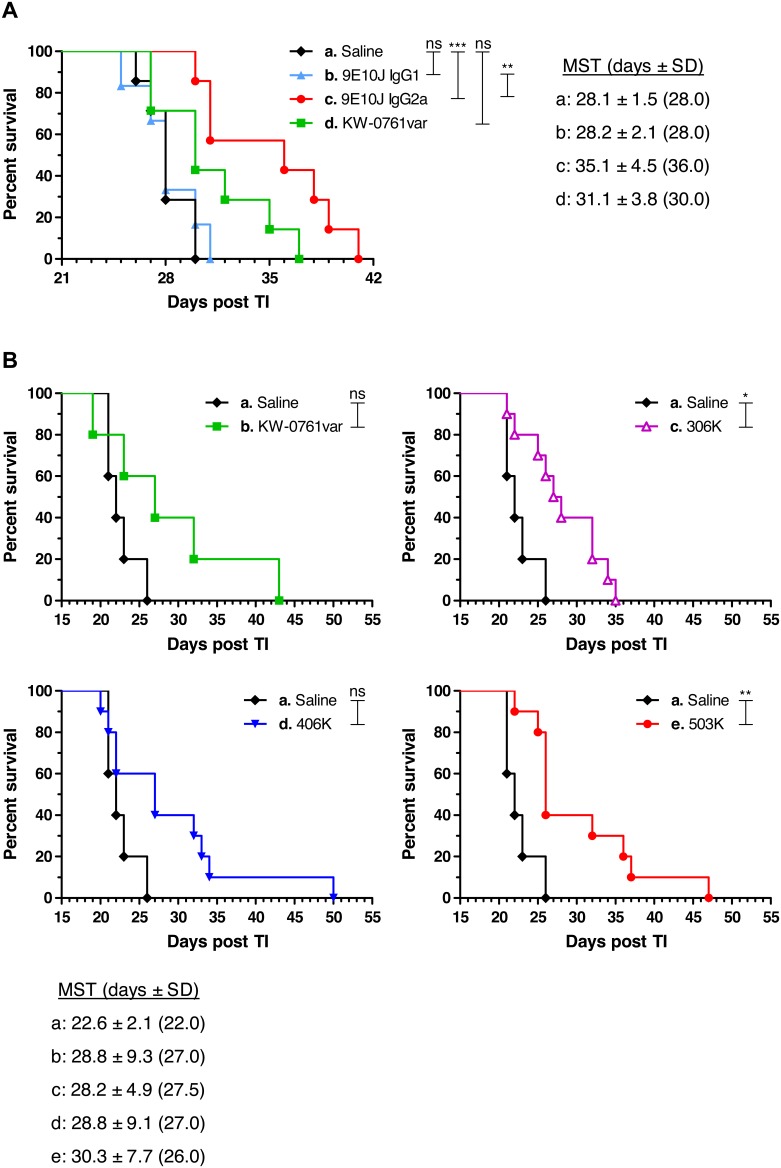
Kaplan-Meier survival plots of nude mice bearing CCRF-CEM tumors. Indicated treatments were given i.v. twice weekly when the tumors became palpable. (**a**) Comparison of different isotypes of anti-CCR4 antibody 9E10J (human IgG1 and murine IgG2a). (**b**) Comparison of affinity improved anti-CCR4 variants 306, 406 and 503 as defucosylated human IgG1. The overlay plots comparing the treated and control groups are shown separately for each treatment group. The mean survival times (*MST*) ± SD for each group are shown in the insets; the median survival times are shown in the brackets. Significant differences between the experimental groups are indicated by *(*P*<0.05), **(*P*<0.01), ***(*P*<0.001), or ns (not significant).

The anti-tumor activity *in vivo* of human anti-CCR4 antibodies was investigated in a xenograft model bearing murine effector system. The mice with pre-established s.c. growing CCRF-CEM tumors were treated with i.v. injections of anti-CCR4 antibodies. In a first experiment, two variants of 9E10J antibody, a fully human IgG1 and a chimeric mouse IgG2a (human variable domains/mouse constant domains) were compared. Data shown in Figure 12a demonstrated that the chimeric 9E10J IgG2a antibody caused an extremely significant survival prolongation (*P*<0.001) in comparison with the control group. However, in this model the fully human IgG1 variant of the same antibody did not show any survival benefit. The defucosylated control antibody KW-0761var showed some survival benefit, although the difference from the control group was not significant (Figure 12a).

In the second experiment, the affinity improved anti-CCR4 antibodies 306, 406 and 503 were compared as defucosylated human IgG1. All the tested antibody variants demonstrated survival prolongation in comparison with the control group, although only the variants 306 and 503 revealed statistically significant difference with the best survival benefit achieved by the treatment with antibody 503 (Figure 12b). In both animal experiments, no effect of antibody treatment on the body weight of the treated animals was found (data not shown).

## Discussion

The present report describes the selection, isolation and characterization of fully human antagonistic antibodies by phage display against GPCR target, the CC chemokine receptor 4. The generation of antibodies against seven-transmembrane G-protein coupled receptors (GPCR) or other multi-spanner membrane proteins is notoriously difficult, due to the lack of suitable antigen reagents (reviewed in [44]). Ideally, the receptor should be pure, homogenous and in a stable conformation similar to that of the native receptor structure. However, this requirement is difficult to fulfil for GPCRs, since the majority of the receptor protein is embedded in the lipid bilayer within the plasma membrane, only the N-terminal domain and the extracellular loops are accessible as potentially immunogenic epitopes.

There are a few examples described in the literature where phage display and the human or mouse antibody libraries (either naïve or semisynthetic) were used for generation of anti-GPCR antibodies. In these examples, the researchers used the surrogate antigens, such as biotinylated linear or cyclic peptides corresponding the N-terminal parts or extracellular loops of GPCR [45]–[48] or GPCR-enriched cell-membrane fractions prepared as paramagnetic proteoliposomes [49], [50].

To overcome the limitations of the pure antigen availability, we used phage display and a cell-based antibody selection (CBAS) approach for generation of fully human antibodies against CCR4. The cell-based panning strategies have been successfully used previously for generating antibodies against bulky membrane antigens, such as EGFR, HER2, ALCAM, EpCAM [51] or c-Met [52]. In general, cell-based screening is often challenging due to the much greater antigen complexity, lower antigen concentration and antigen accessibility. For example, the previous attempts described in the literature to isolate anti-GPCR antibodies by phage display and panning on the cells were unsuccessful [53], [54]. Nevertheless, if successful the whole cell-based selection approaches would allow isolation of antibodies against the membrane-bound receptors in their native environment and natural conformation. In the present study, we demonstrate for the first time in our knowledge that using the transfected cells with high levels of GPCR expression, subtractive library panning and high-throughput screening can lead to isolation of the antibodies specific for the chemokine receptor target.

The initial phage-display derived human anti-CCR4 antibodies were well suited as templates for generation of improved variants. The generated antibodies demonstrated inhibition of ligand induced intracellular signaling and chemotaxis. The signal-antagonizing activity positively correlated with the receptor-binding affinity of the antibody variants and, in addition, was dependent on which CCR4 ligand was used in experiments. For example, the generated anti-CCR4 antibodies were able to completely inhibit intracellular signaling and chemotaxis of CCR4^+^ cells induced by the low affinity ligand CCL17 (*K*_D_, 2.1 nM [55]), while only 70–80% inhibition was observed when the high affinity ligand CCL22 (*K*_D_, 0.18 nM [6]) was used under the same conditions. In addition, the generated anti-CCR4 antibodies possessed strong ADCC activity against CCR4^+^ cells and some tumor cell killing via phagocytosis. They were also able to potently stimulate the killing of autologous CCR4^+^ T_reg_ cells in vitro. The ADCC activity could additionally be enhanced by generation of antibody glycoforms lacking fucose and thus having stronger affinity to FcγRIIIa [56].

The generated anti-CCR4 human antibodies demonstrated ADCC-dependent therapeutic anti-tumor effect *in vivo* in the T-cell deficient nude mice bearing the xenografted human T-cell lymphoma. The animal model used, xenografted human tumor cells in a nu/nu mouse, relies on intact mouse effector cells, such as NK cells, polymorphonuclear leukocytes (PMN) and macrophages. In the first experiment, where the two different isotypes (huIgG1 and muIgG2a) of anti-CCR4 9E10J antibody were compared, the highest anti-tumor activity was observed for the chimeric antibody variant comprising the murine IgG2a Fc portion. Superiority of the muIgG2a isotype over huIgG1 may indicate that the antitumor activity of the antibodies in this model is mainly mediated by mouse PMNs and macrophages [57]. In a second animal experiment, where the different antibody variants were compared (as defucosylated human IgG1), the strongest effect on survival prolongation of the treated mice was demonstrated by the antibodies with the highest affinity to CCR4.

It is known that CCR4 is expressed on human platelets [43] and that the CCR4 ligands, CCL17 and CCL22, can induce platelet activation and aggregation [58], and stimulate platelet adhesion to collagen and fibrinogen [59]. In this report, we showed that the generated anti-CCR4 human antibodies are able to bind to platelets, without affecting their aggregation. In addition, they showed only slight effect on the platelet activation induced by the CCR4 ligands, under the used assay conditions. These data might indicate that the generated anti-CCR4 antibodies could have a favorable safety profile in therapeutic settings with no potential danger of thrombosis or wound healing complications. However, an additional detailed investigation of the antibody effect on platelet function in regard of the chemokine milieu would be needed.

The isolated anti-human CCR4 antibodies were also cross-reactive with the receptors from the non-human primates and mice. The latter feature allowed us to study the immunomodulatory functions of the anti-CCR4 antibodies using syngeneic cancer models in immunocompetent mice (manuscript submitted).

In summary, we have generated a set of fully human and affinity matured antibodies against a chemokine receptor CCR4 and showed their anti-tumor activity both *in vitro* and in an animal model. Unlike the previously described therapeutic antibody KW-0761 (mogamulizumab) [27], [60], the human anti-CCR4 antibodies possess a dual mode of action – inhibition of chemokine-induced signaling/chemotaxis and ADCC/ADCP. In addition, they demonstrate cross-reactivity with the mouse receptor. These findings support further evaluation of our anti-CCR4 antibodies for use in immunotherapy of CCR4^+^ positive tumors and as immunomodulatory agents in different kinds of solid and hematological tumors.

## Supporting Information

File S1 **Contains the files: Table S1 to S4:** Binding inhibition experiments (Table S1 and S2), inhibition of ligand mediated signaling events (Table S3) and ADCC activities (Table S4).
(DOC)
