# Supplementary material for: Fully Human Antagonistic Antibodies against CCR4 Potently Inhibit Cell Signaling and Chemotaxis
Source: PLoS One. 2014 Jul 31;9(7):e103776. doi: 10.1371/journal.pone.0103776 (PMC4117600; doi:10.1371/journal.pone.0103776)
Supplement: File S1 — Contains the files: Table S1 to S4: Binding inhibition experiments (Table S1 and S2), inhibition of ligand mediated signaling events (Table S3) and ADCC activities (Table S4). (DOC) [file pone.0103776.s001.doc]

**Supporting Information**

**Table S1. Apparent affinities (*IC*_50_) as determined from the binding inhibition experiments.**

1. Competition of biotinylated 9E IgG1 (see Figure 5c,d)

|  | ***IC*_50_ (pM) on CCR4^+^ cells** | |
| --- | --- | --- |
| **IgG** | **DT40/CCR4** | **CCRF-CEM** |
| 9E | 440.3 | 377.3 |
| 9E10J | 57.9 | 66.5 |
| Relative potency (*IC*_50_^9E^/*IC*_50_^9E10J^) | 7.6 | 5.7 |

1. Competition of biotinylated 9E10J IgG1 on DT40/CCR4 cells (see Figure 5e)

| **IgG** | ***IC*_50_ (pM)** | **Relative potency (*IC*_50_^9E10J^/*IC*_50_^Ab^)** |
| --- | --- | --- |
| 9E10J | 261.2 | 1 |
| 306 | 76.0 | 3.4 |
| 406 | 56.3 | 4.6 |
| 503 | 61.5 | 4.2 |

1. Competition of labeled ligands on DT40/CCR4 cells (see Figure 5f,g)

| **IgG** | **CCL22** | | **CCL17** | |
| --- | --- | --- | --- | --- |
|  | ***IC*_50_ (pM)** | **Relative potency**  ***IC*_50_^9E10J^/*IC*_50_^Ab^** | ***IC*_50_ (pM)** | **Relative potency**  ***IC*_50_^9E10J^/*IC*_50_^Ab^** |
| 9E10J | 547.6 | 1 | 3,998.0 | 1 |
| 306 | 282.5 | 1.9 | 505.1 | 7.9 |
| 406 | 268.2 | 2.0 | 941.0 | 4.2 |
| 503 | 108.3 | 5.1 | 867.8 | 4.6 |
| CCL22 | 29.8 |  | n.d. |  |
| CCL17 | n.d. |  | 351,700.0 |  |

Labeled antibody variants 9E IgG1 (a) and 9E10J IgG1 (b) as well as the labeled ligands CCL22 and CCL17 (c) competed for binding to CCR4-positive cells in the presence of increasing concentrations of the corresponding unlabeled IgGs or ligands. The resulting competition binding curves were fitted using the ‘log [inhibitor] vs. response’ equation of software PRISM (GraphPad) to determine the *IC*_50_ values. n.d., not determined.

**Table S2. Relative potency of human anti-CCR4 antibodies in blocking ligand-induced signaling.**

1. Comparison of the library-derived antibody 9E and its chain-shuffled derivative 9E10J in antagonizing CCL17-induced Ca^2+^ mobilization (Figure 8). The measurements were performed using FACSCanto II flow cytometer. Means ± SD from three independent experiments are shown.

| **Antibody (IgG1)** | ***IC*_50_ (pM)** | **Relative potency**  ***IC*_50_^9E^/*IC*_50_^9E10J^** |
| --- | --- | --- |
| 9E | 224.8 ± 116.5 | 1 |
| 9E10J | 47.7 ± 4.5 | 4.7 |

1. Comparison of the antibody 9E10J and its mutated variants in antagonizing CCL17-induced Ca^2+^ mobilization (Figure 8j). The measurements were performed using PHERAstar high-throughput microplate reader. Means ± SD from two independent experiments are shown.

| **Antibody (IgG1)** | ***IC*_50_ (pM)** | **Relative potency**  ***IC*_50_^9E^/*IC*_50_^9E10J^** |
| --- | --- | --- |
| 9E10J | 74.0 ± 5.7 | 1 |
| 306 | 38.5 ± 0.7 | 1.9 |
| 406 | 25.5 ± 5.5 | 2.9 |
| 503 | 11.5 ± 2.1 | 6.4 |

1. Comparison of the antibody 9E10J and its mutated variants at a concentration of 10 µg/mL in blocking Ca^2+^ mobilization induced by CCL22 at a concentration of 5 ng/mL. For calculations, CCL22-induced signal (AUC) was set to as 100%.

| **Antibody (IgG1)** | **% Inhibition** | **Relative potency** |
| --- | --- | --- |
| 9E10J | 18 | 1 |
| 306 | 74 | 4.1 |
| 406 | 74 | 4.1 |
| 503 | 64 | 3.6 |

CCR4-positive cells, loaded with the Ca^2+^ sensing dye Fluo-4, were stimulated with the CCR4-specific ligands CCL17 or CCL22, in the presence or absence of antibodies. The release of Ca^2+^ was measured and the resulting AUC value, corresponding to CCR4-receptor activation, was used for determining the relative potency of the antibodies.

**Table S3. Relative potency of human anti-CCR4 antibodies in blocking ligand-induced chemotaxis.**

1. Comparison of the library-derived antibody 9E and its chain-shuffled derivative 9E10J in antagonizing CCL17- or CCL22-induced chemotaxis of CCRF-CEM cells (Figure 9a,b).

| **Antibody (IgG1)** | **CCL17** | | | **CCL22** | | |
| --- | --- | --- | --- | --- | --- | --- |
|  | ***IC*_50_ (nM)** | **Relative potency**  ***IC*_50_^9E^/*IC*_50_^9E10J^** | **Max inhibition (%)** | ***IC*_50_ (nM)** | **Relative potency**  ***IC*_50_^9E^/*IC*_50_^9E10J^** | **Max inhibition (%)** |
| 9E | 21.60 | 1 | 70 | 1.56 | 1 | 27 |
| 9E10J | 2.17 | 9.95 | 100 | 1.35 | 1.16 | 37 |

1. Comparison of the antibody 9E10J and its mutated variants in antagonizing CCL17- or CCL22-induced chemotaxis of CCRF-CEM cells (Figure 9c,d).

| **Antibody (IgG1)** | **CCL17** | | | **CCL22** | | |
| --- | --- | --- | --- | --- | --- | --- |
|  | ***IC*_50_ (nM)** | **Relative potency**  ***IC*_50_^9E10J^/*IC*_50_^Ab^** | **Max inhibition (%)** | ***IC*_50_ (nM)** | **Relative potency**  ***IC*_50_^9E10J^/*IC*_50_^Ab^** | **Max inhibition (%)** |
| 9E10J | 1.13 | 1 | 89.6 | 0.68 | 1 | 40.8 |
| 306 | 0.12 | 9.42 | 99.5 | 0.51 | 1.33 | 76.8 |
| 406 | 0.15 | 7.53 | 98.3 | 1.14 | 0.60 | 78.1 |
| 503 | 0.05 | 22.6 | 95.3 | 54.63 | 0.01 | 63.0 |

The ligand-induced chemotaxis of CCR4-positive CCRF-CEM cells was inhibited by incubation with anti-CCR4 antibodies. The number of migrated cells, counted using a FACSCanto II flow cytometer, was used to calculate both *IC*_50_ values and percentage of inhibition using the samples with the ligand alone as a reference for 100% migration.

**Table S4. Comparative ADCC activity of anti-CCR4 antibodies.**

1. Head-to-head comparison of an affinity-matured variant 9E10J with its parental antibody 9E and with a comparator KM3060 (Figure 10a,b).

| **Antibody** | ***EC*_50_ (nM)** | **Max killing (%)** |
| --- | --- | --- |
| 9E10J | 0.31 | 42.7 |
| 9E | 2.18 | 33.4 |
| 9E10J | 0.05 | 36.0 |
| KM3060var | 0.12 | 27.3 |

1. Effect of kifunensine treatment on ADCC activity (Figure 10c,d).

| **Antibody** | ***EC*_50_ (pM)** | **Max killing (%)** |
| --- | --- | --- |
| 9E10J-K | 15.5 | 69.2 |
| 9E10J | 55.8 | 9.4 |
| KW-0761var | 187.4 | 106.0 |
| KM3060var | 616.2 | 24.5 |

1. Comparison of ADCC activity of affinity matured defucosylated antibodies using three different cell lines (Figure 10e-g).

|  | **CCRF-CEM** | | **L428** | | **HUT78** | |
| --- | --- | --- | --- | --- | --- | --- |
| **Antibody** | ***EC*_50_ (pM)** | **Max killing (%)** | ***EC*_50_ (pM)** | **Max killing (%)** | ***EC*_50_ (pM)** | **Max killing (%)** |
| 306K | 113.7 | 118.0 | 684.8 | 78.6 | 1,019.0 | 41.0 |
| 406K | 99.9 | 121.0 | 676.9 | 69.7 | 1,264.0 | 30.0 |
| 503K | 5.3 | 108.0 | 218.7 | 96.9 | n.d. | n.d. |
| KW-0761var | 315.2 | 69.8 | 54.08 | 75.4 | 422.1 | 51.0 |

CCR4-positive cells were labeled with calcein and incubated with isolated human PBMCs as effector cells in the presence of different concentrations of anti-CCR4 antibodies. Induction of ADCC was expressed as a percentage of lysed cells; the maximal cell lysis was achieved by using detergent and was set as 100%. n.d., not determined.
